# Transcriptomic Analysis of Long-Term Protective Immunity Induced by Vaccination With *Mycoplasma gallisepticum* Strain ts-304

**DOI:** 10.3389/fimmu.2020.628804

**Published:** 2021-02-02

**Authors:** Sathya N. Kulappu Arachchige, Neil D. Young, Anna Kanci Condello, Oluwadamilola S. Omotainse, Amir H. Noormohammadi, Nadeeka K. Wawegama, Glenn F. Browning

**Affiliations:** ^1^Asia-Pacific Centre for Animal Health, Melbourne Veterinary School, Faculty of Veterinary and Agricultural Sciences, The University of Melbourne, Parkville, VIC, Australia; ^2^Department of Veterinary Biosciences, Melbourne Veterinary School, Faculty of Veterinary and Agricultural Sciences, The University of Melbourne, Parkville, VIC, Australia; ^3^Asia-Pacific Centre for Animal Health, Melbourne Veterinary School, Faculty of Veterinary and Agricultural Sciences, The University of Melbourne, Werribee, VIC, Australia

**Keywords:** chicken, *Mycoplasma gallisepticum*, RNA-sequencing, vaccine, ts-304

## Abstract

Live attenuated vaccines are commonly used to control *Mycoplasma gallisepticum* infections in chickens. *M. gallisepticum* ts-304 is a novel live attenuated vaccine strain that has been shown to be safe and effective. In this study, the transcriptional profiles of genes in the tracheal mucosa in chickens challenged with the *M. gallisepticum* wild-type strain Ap3AS at 57 weeks after vaccination with ts-304 were explored and compared with the profiles of unvaccinated chickens that had been challenged with strain Ap3AS, unvaccinated and unchallenged chickens, and vaccinated but unchallenged chickens. At two weeks after challenge, pair-wise comparisons of transcription in vaccinated-only, vaccinated-and-challenged and unvaccinated and unchallenged birds detected no differences. However, the challenged-only birds had significant up-regulation in the transcription of genes and enrichment of gene ontologies, pathways and protein classes involved in infiltration and proliferation of inflammatory cells and immune responses mediated through enhanced cytokine and chemokine production and signaling, while those predicted to be involved in formation and motor movement of cilia and formation of the cellular cytoskeleton were significantly down-regulated. The transcriptional changes associated with the inflammatory response were less severe in these mature birds than in the relatively young birds examined in a previous study. The findings of this study demonstrated that vaccination with the attenuated *M. gallisepticum* strain ts-304 protects against the transcriptional changes associated with the inflammatory response and pathological changes in the tracheal mucosa caused by infection with *M. gallisepticum* in chickens for at least 57 weeks after vaccination.

## Introduction

*Mycoplasma gallisepticum* is the primary etiological agent of chronic respiratory disease (CRD) in chickens. Infection causes inflammation in the upper (1, 2) and lower respiratory tract (3), resulting in tracheal râles, nasal discharge and coughing. The consequent reduced feed consumption, decreased egg production and slower weight gain cause significant economic loss in both egg and meat producing poultry (4–6).

The most effective control measure is maintenance of disease-free flocks by adhering to strict biosecurity, with periodic serological testing and immediate culling of infected flocks (7). Administration of antimicrobial drugs in the feed or water and vaccination are effective approaches to control of disease in those large-scale commercial poultry farms in which maintenance of disease-free flocks is not feasible (8). Inactivated vaccines reduce clinical signs of the disease and production losses, but offer minimal protection against infection (9). Several live attenuated vaccines, including F strain (7), strain 6/85 (10) and ts-11 (11) are used to immunize chickens against *M. gallisepticum* and have been shown to have greater efficacy in prevention of mycoplasmosis than killed vaccines (12).

Attachment of *M. gallisepticum* to target epithelial cells is essential for colonization of the respiratory mucosa of the host. This interaction is partially mediated by a primary cytadhesin (Gap A) (13, 14) and cytadhesin-related molecules (Crm), including CrmA (15). The level of protection induced by vaccines is thought to depend on successful attachment to respiratory epithelial cells and subsequent colonization (12). The live attenuated *M. gallisepticum* vaccine strain ts-11 (Vaxsafe MG, Bioproperties Australia Pty. Ltd., Glenorie, NSW, Australia) is a temperature sensitive (ts) mutant generated by chemical mutagenesis. Administration of a single dose by eye-drop inoculation achieves colonization of the upper respiratory tract that persists for extended periods, inducing long-term immunity in chickens (16, 17). However, the protective immunity offered by this vaccine is highly dose-dependent (18) and serum antibody responses in chickens vaccinated with ts-11 can be highly variable (19). These characteristics of the immunity induced by vaccination with ts-11 are likely to be due to the presence of two variants in the commercial Vaxsafe MG (ts-11) vaccine, one with an intact *gapA* gene (GapA^+^) and a second with a 20 bp repeat in the *gapA* gene (GapA^-^) that induces a frameshift and results in premature termination of translation of the gene (20). A GapA^+^ clone, strain ts-304, has been isolated from ts-11 and has been shown to be as safe in chickens as the ts-11 strain from which it was derived, and as effective, but at a lower dose (21, 22). The ts-304 vaccine has also been shown to colonize the trachea of turkeys and protect against experimental challenge with the *M. gallisepticum* wild-type strain Ap3AS (23, 24).

Transcriptomic analyses have been used to examine the maladaptive host responses seen after acute (25–27) and chronic (28) infection with *M. gallisepticum*, and to examine the protective host responses of chickens vaccinated with live attenuated strains, including strains GT5 and Mg7 (26). A previous study by our laboratory examined the protective immunity afforded by vaccination with strain ts-304 against chronic *M. gallisepticum* infection, but only at four weeks after vaccination (28). In a recent study we examined the duration of immunity induced by vaccination with ts-304 and found that it persisted for at least 57 weeks after a single vaccination at 3 weeks of age (29).

In order to further understand the protective immunity offered by this new vaccine strain, we explored the transcriptional profiles of the tracheal mucosa in chickens challenged with the *M. gallisepticum* wild-type strain Ap3AS at 57 weeks after vaccination and compared these to the profiles of unvaccinated chickens of a similar age that had also been infected with strain Ap3AS.

## Materials and Methods

### Experimental Vaccination and Infection of Chickens

The procedures used for experimental vaccination and infection with virulent *M. gallisepticum* have been described previously (29). Briefly, 20 White Leghorn specific-pathogen-free (SPF) chickens were randomly allocated into two different groups of 10 (Groups 1 and 2) and inoculated by eye-drop with 30 μl ts-304 vaccine containing 10^6.0^ color changing units (CCU) of strain ts-304 at 3 weeks of age and kept in microbiologically secure isolators until they were 60 weeks of age. Another fifteen control birds were obtained from the SPF flock close to the time of experimental challenge and divided into two groups, a challenged-only positive control group (Group 3), containing 10 birds, and an uninfected negative control group (Group 4), containing 5 birds. Fifty-seven weeks after vaccination, birds from Groups 1 (vaccinated-and-challenged) and 3 (challenged-only) were exposed to an aerosol of the *M. gallisepticum* wild-type strain Ap3AS using an established protocol (22, 30). The birds in Groups 3 and 4 were 70 weeks of age at the time of experimental challenge. Two weeks after challenge all birds were humanely euthanized and necropsied.

### Total RNA Extraction

Total RNA was extracted from the tracheal mucosae of three randomly chosen birds from each group. Briefly, tracheal mucosae were separated from tracheal tissue sections of individual birds using sterile forceps. Approximately 20 mg of mucosal tissue from each bird was disrupted and homogenized in 600 µl of RLT buffer (RNeasy Mini Kit, QIAGEN, Hilden, Germany) using Discofix 3-way stopcocks (B. Braun, Melsungen, Germany). Total RNA was extracted from each tracheal mucosal homogenate using an RNeasy Mini Kit according to the manufacturer’s instructions. All the RNA elutes were treated with DNase using the TURBO DNA-*free* kit (Invitrogen, Carlsbad, CA, USA) and then cleaned and concentrated using the Zymo RNA Clean & Concentrator-25 (Zymo research Corporation, Irvine, CA, USA) kit as recommended by the manufacturers. The quality of the RNA was confirmed using the Agilent 4200 TapeStation system (Agilent Technologies, Santa Clara, CA, USA) and samples with RNA integrity numbers (RIN) of > 8 were further processed for sequencing.

### Illumina Sequencing

The cDNA libraries were constructed using a TrueSeq Stranded mRNA library preparation kit (Illumina Inc., San Diego, CA, USA) according to the manufacturer’s instructions. Briefly, mRNA was separated from 300 to 500 ng of total RNA from each trachea using Oligo dT magnetic beads. First strand cDNA was synthesized from purified, fragmented mRNA using reverse transcriptase and random hexamer primers. The second strand was then synthesized using dUTP, dATP, dCTP, dGTP, DNA polymerase and RNase, and amplified by PCR. The end products were purified after end-repair and ligation of adapters, and the fragment size (~260 bp) and the quality of the cDNA libraries were confirmed using the Agilent 2200 TapeStation system. The libraries were normalized to 1 nM and then pooled, denatured and sequenced on a NextSeq500 sequencing platform (Illumina) to obtain 150 bp paired-end reads.

### Quality Control and Pre-processing of RNA-seq Reads

The quality of raw reads was assessed using FastQC (version 0.11.8, https://www.bioinformatics.babraham.ac.uk/projects/fastqc/). Illumina sequencing adapter sequences and bases with a PHRED quality score of ≤ 20 were removed using Trim Galore (version 0.6.4, https://www.bioinformatics.babraham.ac.uk/projects/trim_galore/), and BBMap (version 38.73, https://sourceforge.net/projects/bbmap/) was used to remove non-paired reads. The gff3, gtf, and fasta files of the annotated *Gallus gallus* (chicken) genome were downloaded from the Ensembl database release 98 (31) and the mRNA sequences for protein-coding genes were extracted, indexed and used for high quality paired read RNA-seq mapping (32) using bowtie2 version 2.3.5 (33). The expected read count per gene was calculated using RSEM version 1.3.2 (32).

### Differential Gene Transcription Analysis

Differential gene transcription analysis was performed in the RStudio environment (version 1.2.5019, http://www.rstudio.com/) with expected counts per gene as the input data using the limma version 3.40.6 (34), Glimma version 1.12.0 (35) and edgeR version 3.26.8 (36) R/Bioconductor packages following recommended guidelines (37). Briefly, read counts were first converted into counts-per-million (CPM) to standardize for differences in library size. Genes with low transcription at a threshold CPM value of ≤ 1 in ≥ 3 samples were excluded from further analysis. Filtered data were normalized for differences in distributions of transcripts between samples using the trimmed mean of M-values (TMM) method (38). Heteroscedascity was removed from count data using voom (39) and CPMs were log_2_ transformed. The mean-variance relationship was accommodated using precision weights calculated using the voom function (39) and a linear model was fit to the normally distributed log_2_ CPM values to compare the gene transcription between the groups. The genes that had a log_2_ (1) fold change (i.e. 2-fold) in gene transcription at a false discovery rate (FDR) of < 0.01 were considered significant. Pairwise comparisons were performed between: the challenged-only group and the negative control group; the challenged-only group and the vaccinated-and-challenged group; the challenged-only group and the vaccinated-only group; the vaccinated-and-challenged group and the negative control group; the vaccinated-and-challenged group and the vaccinated-only group; and the vaccinated-only group and the negative control group. Differentially transcribed genes are reported as up- or down-regulated in the first group compared to the second group in each comparison.

### Enrichment of Genes Within Gene Ontology Categories

Differentially transcribed genes were further analyzed by gene ontology (GO) enrichment using the topGO version 2.36.0 (40) R/Bioconductor package as previously described (28). Briefly, a *G. gallus* GO universe was constructed using GO-IDs linked to each protein-coding gene of *G. gallus*, extracted using the biomaRt annotation tool version 2.40.0 (41), to identify molecular functions (MFs), biological processes (BPs), and cellular components (CCs) enriched with up- or down-regulated genes at a FDR of < 0.01. GO terms significantly enriched with up- or down-regulated genes were further summarized by removing redundant GO terms using the REVIGO web server (42).

### Pathway and Protein Class Analysis

Identification of biological pathways and protein classes enriched with up- or down-regulated genes was performed using the Reactome and Panther databases available in the PANTHER classification tool version 14.1 (43). Pathways and protein classes enriched at a FDR of < 0.01 were reported as significant.

## Results

### The Transcriptional Profiles of the Vaccinated Birds Were Similar to Those of the Uninfected Birds

**Figure 1:**
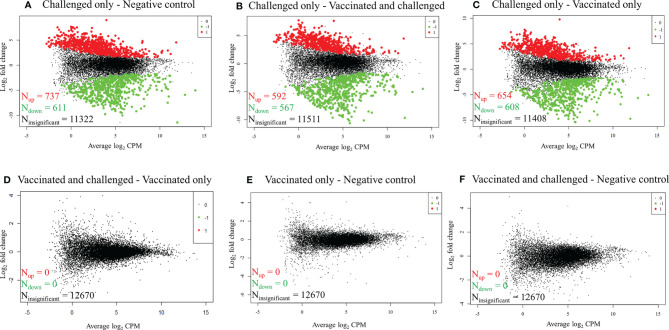
Mean-difference plots of log_2_ fold change versus log_2_ CPM. **(A)** Challenged-only versus negative control birds. **(B)** Challenged-only versus vaccinated-and-challenged birds. **(C)** Challenged-only versus vaccinated-only birds. **(D)** Vaccinated-and-challenged versus vaccinated-only birds. **(E)** Vaccinated-only versus negative control birds. **(F)** Vaccinated-and-challenged versus negative control birds. N indicates number of genes, red dots and numbers indicate up-regulated genes, green dots and numbers indicate down-regulated genes and black dots and numbers represent the genes with no significant difference in transcription between the groups in each comparison.

RNA-seq reads obtained from each sample were mapped to 16,779 *G. gallus* protein-coding genes and 12,670 genes with a CPM of > 1 in three or more samples were retained for differential gene transcription analysis across the four groups. At a FDR of < 0.01, 1,348/12,670 (10.64%) genes were differentially transcribed in the challenged-only group compared to the negative control group, with more genes up-regulated (737/1,348, 54.67%) than down-regulated (611/1,348, 45.33%) (**Figure 1A**). Similarly, 1,159/12,670 (9.15%) genes had significant differences in transcription in the challenged-only group compared with the vaccinated-and-challenged group, with more genes up-regulated (592/1,159, 51.08%) than down-regulated (567/1,159, 48.92%) (**Figure 1B**). A total of 1262/12670 (9.96%) genes had significant differences in transcription in the challenged-only group compared with the vaccinated-only group, with more genes up-regulated (654/1,262, 51.82%) than down-regulated (608/1,262, 48.18%) (**Figure 1C**). There were no detectable differences in gene transcription between the vaccinated-only group and the vaccinated-and-challenged or negative control groups (**Figures 1D, E****)**. Similarly, no gene transcription differences were detected between the vaccinated-and-challenged and the negative control group (**Figure 1F**).

### Most Differences in Transcription between the Challenged-only Group and the Other Groups Were Shared

**Figure 2:**
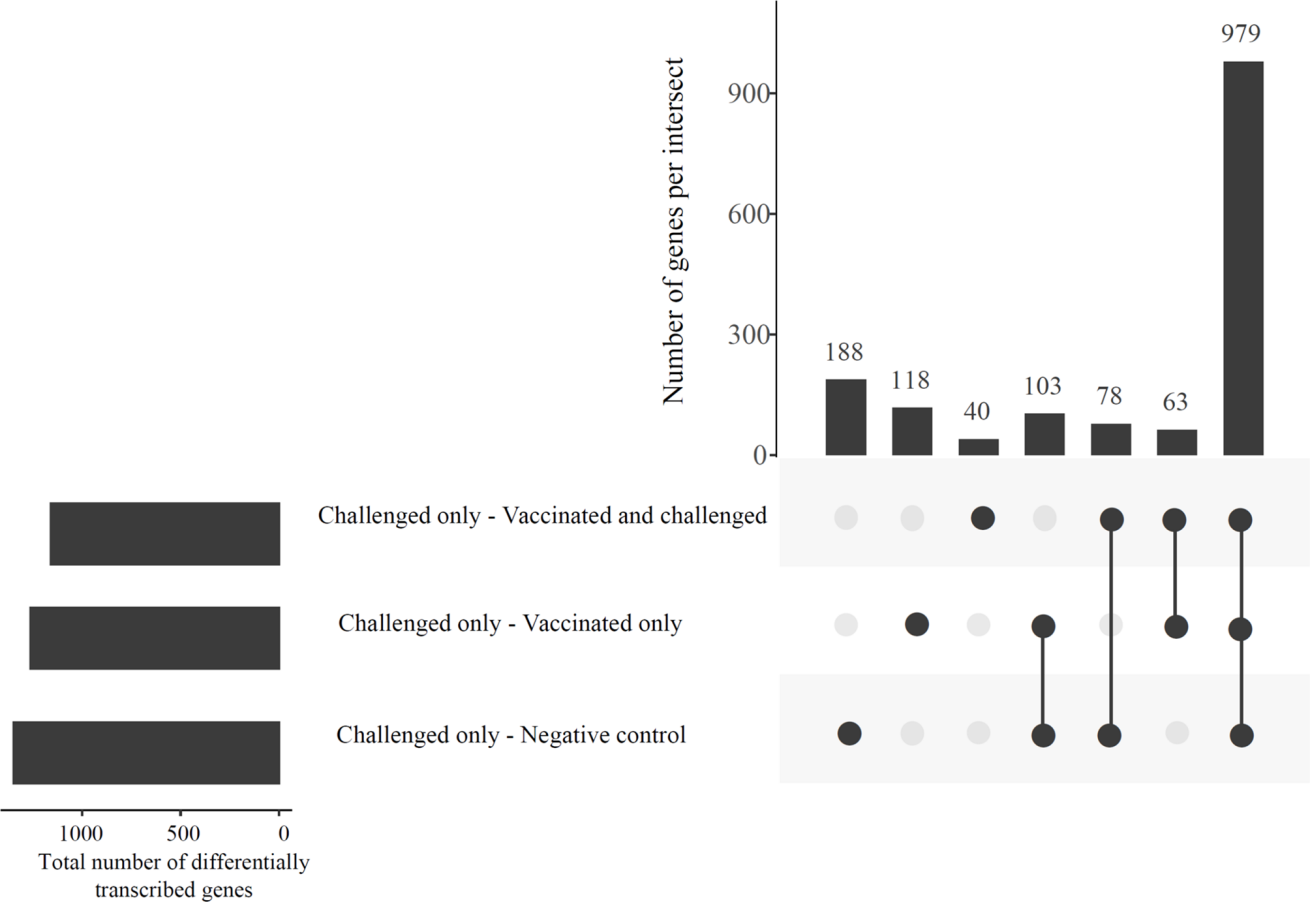
An UpSetR plot displaying the abundances of common and unique significantly differentially transcribed genes in the challenged-only group compared to the negative control, vaccinated-and-challenged and vaccinated-only groups. Horizontal bars indicate the total number of differentially transcribed genes in each comparison.

The gene transcription analysis detected 1,568 genes that were differentially transcribed in one, two or all comparisons between the challenged-only group and the other three groups (negative control, vaccinated-only and vaccinated-and-challenged) while no significant differences in transcription were detected for 11,102 genes in these comparisons (**Figure 2**). Of the 1,568 (62.44%) differentially transcribed genes, 979 were differentially transcribed in all three comparisons (**Figure 2**). A further 78 differentially transcribed genes were common to the comparisons of the challenged-only and negative control groups and the challenged-only and vaccinated-and-challenged groups. Another 63 differentially transcribed genes were common to comparisons of the challenged-only and vaccinated-and-challenged groups and the challenged-only and vaccinated-only groups, and 103 differentially transcribed genes were common to comparisons of the challenged-only and vaccinated-only groups and the challenged-only and negative control groups. There were 188 differentially transcribed genes unique to comparisons of the challenged-only and negative control groups, 40 unique to comparisons of the challenged-only and vaccinated-and-challenged groups, and 118 unique to comparisons of the challenged-only and vaccinated-only groups (**Figure 2**).

### The Most Up-Regulated Genes in the Challenged-Only Group Compared to the Other Groups Mainly Encode Proteins Involved in DNA Replication and the Cell Cycle

The 25 genes that were most differentially regulated in the challenged-only group compared to each of the other groups based on FDR included 16 genes that were up-regulated compared to the negative control group, 7 up-regulated compared to the vaccinated-and-challenged group and 9 up-regulated compared to the vaccinated-only group (**Table 1**). Among these most up-regulated genes, TXNDC5 (thioredoxin domain-containing protein 5) had the most significant difference in transcription between the challenged-only group and all the other three groups. Among these most up-regulated genes in the challenged-only group were 12 encoding proteins involved in DNA replication and the cell cycle that were up-regulated compared to the negative control group, 5 encoding proteins involved in DNA replication and the cell cycle that were up-regulated compared to the vaccinated-and-challenged group, and 7 encoding proteins involved in DNA replication and the cell cycle that were up-regulated compared to the vaccinated-only group. One gene encoding a protein involved in prevention of apoptosis and another encoding a protein involved in degradation of the extracellular matrix were also among the most up-regulated genes compared to all the other groups (**Table 1**).

**Table 1:** Log_2_ fold changes (FC) and false discovery rates (FDR) of **t**he up-regulated genes among the 25 most differentially expressed genes based on FDR in the challenged-only group compared to the other groups.

Gene name	Challenged-only group compared to:					
	Negative control		Vaccinated-and-challenged		Vaccinated-only	
	FC	FDR	FC	FDR	FC	FDR
**DNA replication and cell cycle**						
Cell division cycle associated 3	5.931	6.49E-06	5.673	6.57E-06	6.045	5.57E-06
Sperm associated antigen 5	5.362	6.49E-06	5.246	9.89E-06	5.840	5.63E-06
Ubiquitin conjugating enzyme E2 C	5.274	6.49E-06	4.613	8.70E-06	5.448	5.48E-06
Karyopherin subunit alpha 2	4.866	5.03E-06	4.774	4.22E-06	4.863	4.49E-06
Minichromosome maintenance complex component 5	4.480	6.49E-06	4.304	8.70E-06	4.654	5.57E-06
Thymidine kinase 1	5.280	6.49E-06			6.243	5.73E-06
Topoisomerase (dna) II alpha	4.792	6.72E-06			5.400	5.73E-06
Cyclin B3	4.842	7.46E-06				
Myb proto-oncogene-like 2	4.260	6.72E-06				
Cyclin B1	4.133	6.49E-06				
Minichromosome maintenance complex component 2	3.770	9.19E-06				
Ribonucleotide reductase regulatory subunit m2	6.196	6.49E-06				
						
**Prevention of apoptosis**						
Thioredoxin domain containing 5	4.476	8.93E-08	4.371	1.50E-07	4.291	1.81E-07
						
**Other**						
Matrix metallopeptidase 7	9.003	6.12E-06	7.630	3.51E-06	9.789	5.63E-06
Biliverdin reductase A	5.808	8.52E-06				
Derlin 3	3.006	6.49E-06				

A false discovery rate (FDR) of < 0.01 was considered significant.

### The Most Down-Regulated Genes in the Challenged-Only Group Compared to Other Groups Mainly Encode Proteins Involved in Formation and Motor Movement of the Cilia

The 25 genes that were most differentially regulated in the challenged-only group compared to each of the other groups based on FDR included 9 that were down-regulated compared to the negative control group, 18 that were down-regulated compared to the vaccinated-and-challenged group and 16 that were down-regulated compared to the vaccinated-only group (**Table 2**). The PACRG (Parkin coregulated) gene, which is involved in assembly and motor movement of the cilia, had the greatest difference in transcription between the challenged-only group and all the other three groups (**Table 2**). Among these most down-regulated genes in the challenged-only group were 3 genes involved in formation and motor movement of cilia that were down-regulated compared to the negative control group and 6 involved in formation and motor movement of cilia that were down-regulated compared with both the vaccinated-and-challenged and vaccinated-only groups. In addition, these most down-regulated genes in the challenged-only group included 1 gene involved in oxidoreductase activity that was down-regulated compared to the negative control group, 3 involved in oxidoreductase activity that were down-regulated compared to the vaccinated-and-challenged group, and 2 involved in oxidoreductase activity that were down-regulated compared to the vaccinated-only group. In addition, this group of most down-regulated genes included two genes involved in metabolism that were down-regulated compared with the negative control and vaccinated-only groups.

**Table 2:** Log_2_ fold changes (FC) and false discovery rates (FDR) of **t**he down-regulated genes among the 25 most differentially expressed genes based on FDR in the challenged-only group compared to the other groups.

Gene name	Challenged-only group compared to:					
	Negative control		Vaccinated-and-challenged		Vaccinated-only	
	FC	FDR	FC	FDR	FC	FDR
**Assembly and motor movement of cilia**						
Parkin coregulated	−7.674	5.03E-06	−8.529	1.99E-06	−8.416	2.14E-06
PIH1 domain containing 3	−5.313	5.03E-06	−5.713	2.04E-06	−5.695	2.14E-06
UBX domain protein 10	−4.678	6.49E-06	−4.999	3.51E-06	−4.969	4.49E-06
Enkurin, TRPC channel interacting protein			−6.618	4.22E-06	−6.292	5.63E-06
Doublecortin domain-containing 2B			−5.470	6.57E-06	−5.544	5.57E-06
Dynein assembly factor with WD repeats 1			−5.272	8.16E-06	−5.379	5.63E-06
						
**Oxidoreductase activity**						
Glutathione S-transferase omega 2			−3.523	3.51E-06	−3.670	3.46E-06
Peroxiredoxin 6			−3.073	3.51E-06	−2.789	5.57E-06
Aldo-keto reductase family 1, member B1-like			−3.843	1.01E-05		
Cytochrome P450 2G1-like	−5.914	6.49E-06				
						
**Metabolism**						
Adenylate kinase 8	−7.045	5.93E-06			−7.227	5.17E-06
Serine/threonine/tyrosine interacting-like 1	−5.694	4.22E-06			−5.782	4.49E-06
						
**Other**						
EF-hand calcium binding domain 10	−5.329	6.49E-06	−5.930	3.51E-06	−6.012	3.46E-06
Coiled-coil domain containing 27	−5.259	6.49E-06	−5.787	3.51E-06	−5.931	3.46E-06
Chromosome 11 open reading frame 88			−8.748	1.01E-05	−8.924	7.17E-06
MORN repeat containing 3			−5.808	7.28E-06	−5.860	5.63E-06
Chromosome 9 open reading frame 116			−5.171	3.51E-06	−5.056	4.49E-06
Transmembrane protein 68-like	−6.727	6.49E-06			−6.482	6.66E-06
Uncharacterized loc107050476	−4.878	6.49E-06	−4.728	8.16E-06		
Aquaporin 5	−4.603	9.19E-06				
Chromosome 3 open reading frame 67			−6.029	1.03E-05		

A false discovery rate (FDR) of < 0.01 was considered significant.

### Challenged-Only Birds Had Altered Transcription of Genes That Drive Immune Responses

As *M. gallisepticum* is known to cause immune dysregulation in younger birds, further analyses of immune response-related genes were conducted. The differentially expressed cytokine, chemokine and cytokine/chemokine receptor genes are shown in **Table 3**. The gene for interferon-γ (IFN-γ) was up-regulated, along with that for interleukin (IL)-4–induced 1, in the challenged-only group compared to all the other three groups. The IL-4–induced 1 gene had the greatest log_2_ fold changes (FCs), with a FC of 7.69 between the challenged-only and negative control groups, 7.71 between the challenged-only and vaccinated-and-challenged groups, and 7.27 between the challenged-only and vaccinated-only groups. Genes for several cytokines, including IL-16, were up-regulated in the challenged-only group compared to the negative control and vaccinated-only groups, while the IL-22 gene was up-regulated in the challenged-only group only in comparison with the negative control group. A number of genes for chemokines, including those for C-C motif chemokine ligand (CCL)-26, CCL-19, C-X-C motif chemokine ligand (CXCL)13, CXCL13-like 2, CXCL13-like 3, and chemokine ah221 (chCCLi9), were up-regulated in the challenged-only group compared to negative control group (with FCs ranging from 4.41 to 6.70), the vaccinated-and-challenged group (with FCs ranging from 3.99 to 6.38), and the vaccinated-only group (with FCs ranging from 5.23 to 6.73). The genes for the chemokines CCL-4 (macrophage inflammatory protein (MIP)-1β), CXCL12, and X-C motif chemokine ligand (XCL)1/lymphotactin were up-regulated in the challenged-only group compared to both the negative control and vaccinated-only groups. The gene for the CXCL1-like chemokine was up-regulated in the challenged-only birds only in comparison with the negative control birds. The gene for CCL-20 (MIP-3α) was the only chemokine gene that was down-regulated in the challenged-only group, with a FC of −3.90, and it was differentially transcribed only in comparison with the vaccinated-and-challenged group. In addition, there were several genes for cytokine and chemokine receptors that were differentially transcribed between the groups included in the three comparisons. Even though the genes for IL-1α and IL-1β were not differentially transcribed, the gene for IL-1 receptor-like 2 was down-regulated in the challenged-only group compared to the negative control group and the vaccinated-only group, while the gene for IL-1 receptor associated kinase 1 binding protein 1 was down-regulated in the challenged-only group compared to the vaccinated-and-challenged group and the vaccinated-only group.

**Table 3:** Log_2_ fold changes (FC) and false discovery rates (FDR) of differentially expressed genes for cytokines, chemokines and their receptors in the challenged-only group compared to the other groups.

Gene name	Challenged-only group compared to:					
	Negative control		Vaccinated-and-challenged		Vaccinated-only	
	FC	FDR	FC	FDR	FC	FDR
**Cytokines**						
Interleukin-4 induced 1	7.686	2.77E-04	7.712	5.19E-04	7.27	3.40E-04
Interferon gamma	5.982	1.87E-04	6.049	3.18E-04	6.918	1.83E-04
Interleukin-22	5.109	3.23E-03				
Interleukin-16	3.438	2.53E-03			3.545	2.87E-03
						
**Chemokines**						
C-C motif chemokine ligand 26	6.703	1.08E-05	6.378	2.08E-05	6.289	1.38E-05
C-C motif chemokine ligand 19	5.193	9.54E-05	5.635	1.50E-04	5.269	1.23E-04
C-X-C motif chemokine ligand 13-like 3	5.196	6.51E-04	4.23	2.59E-03	5.681	6.34E-04
C-X-C motif chemokine ligand 13	5.453	8.57E-04	5.161	1.72E-03	5.751	9.87E-04
C-X-C motif chemokine ligand 13-like 2	7.313	3.26E-03	7.307	5.67E-03	6.725	4.42E-03
Chemokine ah221	4.412	2.97E-03	3.996	7.05E-03	5.232	2.02E-03
C-C motif chemokine ligand 4 (MIP-1β)	3.23	5.87E-03			4.108	2.40E-03
C-X-C motif chemokine ligand 12	2.49	2.88E-03			2.253	8.76E-03
X-C motif chemokine ligand 1	3.956	9.41E-03			4.934	5.32E-03
Chemokine (C-X-C motif) ligand 1-like	5.079	2.80E-03				
C-C motif chemokine ligand 20			−3.903	9.76E-03		
						
**Cytokine receptors**						
Interleukin-2 receptor subunit gamma	5.084	9.19E-06	4.449	2.24E-05	4.772	1.28E-05
Interleukin-12 receptor subunit beta 1	6.604	2.74E-04	5.647	8.08E-04	5.852	5.20E-04
Interleukin-21 receptor	4.736	9.26E-04	4.145	2.86E-03	4.684	1.25E-03
Interleukin-20 receptor subunit alpha	3.236	6.20E-04	2.844	2.57E-03	3.187	8.23E-04
Interleukin-13 receptor subunit alpha 2	4.778	2.18E-03	3.899	7.70E-03	4.845	3.07E-03
Interleukin-7 receptor	2.71	9.87E-03	3.113	6.97E-03	2.925	7.61E-03
Interleukin-10 receptor subunit alpha	2.64	4.56E-03	3.017	2.87E-03	2.832	3.52E-03
Colony stimulating factor 3 receptor	3.89	2.66E-04	3.474	8.88E-04	3.595	5.05E-04
TNF receptor superfamily member 13B	5.724	1.22E-04	4.846	3.22E-04	4.852	2.38E-04
Granulocyte-macrophage colony-stimulating factor receptor subunit alpha-like	3.56	1.34E-03	3.583	2.43E-03	3.169	3.54E-03
Colony stimulating factor 2 receptor beta common subunit	3.166	5.23E-03	3.487	3.97E-03	3.727	2.46E-03
TNF receptor superfamily member 1B	2.853	3.85E-03	2.697	9.67E-03	2.918	4.36E-03
Cytokine receptor like factor 3	2.235	5.10E-03	2.327	6.03E-03		
Interleukin 1 receptor-like 2	−2.439	2.35E-03			−2.19	9.04E-03
TNF receptor superfamily member 4	3.418	2.13E-03			2.889	7.83E-03
Interleukin 1 receptor associated kinase 1 binding protein 1			−2.765	6.43E-04	−2.73	6.28E-04
Cytokine receptor common subunit beta-like	3.291	6.79E-03				
						
**Chemokine receptors**						
C-X-C chemokine receptor type 3-like	−3.403	2.21E-04	−3.169	5.74E-04	−3.827	6.99E-05
X-C motif chemokine receptor 1	5.757	3.78E-04	5.4	6.67E-04	5.144	6.74E-04
C-C motif chemokine receptor 10	7.524	8.90E-05	6.273	1.50E-04	6.423	1.15E-04
C-X-C motif chemokine receptor 5	3.951	4.18E-03	5.012	3.10E-03	4.68	2.67E-03
C-X-C motif chemokine receptor 4	3.183	5.19E-04	3.813	3.46E-04	3.212	6.27E-04
C-X3-C motif chemokine receptor 1	4.414	2.13E-03	3.572	9.53E-03	3.909	4.68E-03
C-C motif chemokine receptor 2	2.936	6.70E-03	3.911	2.60E-03		
C-C motif chemokine receptor 5	6.12	4.71E-05	5.555	1.04E-04		

A false discovery rate (FDR) of < 0.01 was considered significant.

Genes for several Toll-like receptors (TLRs), including those for TLR-7, TLR-2 family member B and TLR-1 family member A, were up-regulated in the challenged-only group compared with all three other groups. TLR-5 was the only down-regulated TLR in the challenged-only group and only in comparison with the vaccinated-only group (**Table 4**). Five major histocompatibility complex (MHC) class II-related protein-coding genes, and two MHC class I antigen-related protein genes, were among the differentially transcribed genes. In addition, several immunoglobulin (Ig)-related protein genes, including 2 genes for Fc fragment linked proteins, 2 genes for Ig superfamily members and 2 genes for osteoclast-associated Ig-like receptor-like proteins, and several complement-related protein genes, including 5 genes for complement C1 linked proteins, were differentially transcribed in the challenged-only group compared to one or more of the other groups (**Table 4**).

**Table 4:** Log_2_ fold changes (FC) and false discovery rates (FDR) of differentially expressed MHC and other immune response-related genes in the challenged-only group compared to the other groups.

Gene name	Challenged-only group compared to:					
	Negative control		Vaccinated-and-challenged		Vaccinated-only	
	FC	FDR	FC	FDR	FC	FDR
**MHC antigen-related genes**						
Major histocompatibility complex, class II, DM alpha	4.083	4.46E-05	2.507	1.94E-03	3.402	1.48E-04
Major histocompatibility complex class I antigen BF2	2.605	6.78E-04	2.298	3.10E-03	2.6	7.01E-04
Major histocompatibility complex, class II, DM beta 2	4.015	2.50E-04	2.842	3.51E-03	3.087	1.40E-03
Major histocompatibility complex class II beta chain BLB2	2.824	8.01E-04			2.591	2.03E-03
Major histocompatibility complex class II beta chain BLB1	3.544	3.12E-03			3.319	5.93E-03
Major histocompatibility complex, class II, DM beta 1	5.857	2.08E-04			3.321	4.18E-03
Major histocompatibility complex, class I, A6					2.183	1.25E-03
						
**Immunoglobulin-related genes**						
Fc receptor-like protein 3-like	4.575	4.10E-04	4.637	6.61E-04	6.047	2.07E-04
V-set and immunoglobulin domain containing 4	2.717	3.01E-03	2.551	7.70E-03		
Immunoglobulin superfamily member 1-like	6	1.37E-03			4.617	6.14E-03
Immunoglobulin superfamily member 1	3.634	4.22E-03				
Osteoclast-associated immunoglobulin-like receptor-like	3.68	6.47E-03				
Osteoclast-associated immunoglobulin-like receptor	3.719	8.74E-03				
Fc fragment of IgE receptor Ig	4.237	8.44E-03				
Immunoglobulin-like receptor CHIR-B3					3.568	9.53E-03
						
**Toll-like receptors**						
Toll-like receptor 7	4.904	1.06E-03	4.306	3.40E-03	4.463	1.93E-03
Toll-like receptor 2 family member B	5.489	2.59E-03	5.295	4.82E-03	4.56	6.14E-03
Toll-like receptor 1 family member A	3.406	3.42E-03	3.597	4.83E-03	5.117	7.89E-04
Toll-like receptor 5					−2.747	3.54E-03
						
**Complement-related genes**						
Complement C3a receptor 1	3.713	6.51E-04	4.005	9.40E-04	3.136	2.33E-03
Complement C1s	2.761	8.30E-04	2.913	8.11E-04	2.806	8.26E-04
Complement C1q A chain	3.028	1.53E-03	2.547	8.67E-03	2.761	3.83E-03
Complement component 3	1.869	4.94E-03	2.063	2.06E-03	2.566	1.61E-04
Complement 4	2.554	5.74E-03	2.555	8.69E-03	2.578	6.80E-03
Complement C1q C chain	3.131	2.14E-03			2.624	9.25E-03
Complement C1q B chain	3.52	3.27E-03				
Complement C1q binding protein	1.755	1.23E-03				
Complement component 4					2.098	8.14E-03
Complement C7					3.353	9.07E-03

A false discovery rate (FDR) of < 0.01 was considered significant.

### Gene Ontologies (GOs) Enriched With Up-Regulated Genes in the Challenged-Only Group Were Mainly Genes for the Immune Response, DNA Replication, Cell Activation and Mitosis

Functional GO analysis enabled the identification of cellular components, molecular functions and biological processes enriched with up- or down-regulated genes. Fifty one cellular components, 55 molecular functions, and 439 biological processes were significantly enriched with up-regulated genes in the challenged-only group compared to the negative control group, while 39 cellular components, 53 molecular functions, and 319 biological processes were enriched with up-regulated genes in the challenged-only group compared to the vaccinated-and-challenged group, and 42 cellular components, 59 molecular functions, and 393 biological processes were enriched with up-regulated genes in the challenged-only group compared to the vaccinated-only group. The REVIGO non-redundant functional GO summaries of the challenged-only group compared to the negative control, vaccinated-and-challenged, and vaccinated-only groups had similar profiles in the Cellular Component, Molecular Function, and Biological Process GO categories. In brief, the cellular components enriched with up-regulated genes included intracellular components, including those associated with the chromosome, the microtubule spindle and the cyclin-dependent protein kinase holoenzyme complex, which are involved in mitosis, as well as cell surface components involved in ligand-receptor binding (**Figure S1**). The molecular functions enriched with up-regulated genes were associated with DNA helicase activity, single-stranded DNA binding, protein binding, particularly antigen, Ig, cytokine and chemokine binding, signal transduction, chemokine activity and cytokine-receptor activity (**Figure S2**). The biological processes enriched with up-regulated genes were associated with DNA replication and the mitotic cell cycle, immune responses mediated by cytokines and chemokines, and activation of leukocytes (**Figure S3**). The cellular component with the greatest proportion of up-regulated genes per GO term in the challenged-only group compared to the negative control, vaccinated-and-challenged and vaccinated-only groups was the condensed nuclear chromosome kinetochore, with 75% (9/12) of genes up-regulated. The molecular functions with the greatest proportion of up-regulated genes per GO term in the challenged-only group were ATP-dependent DNA helicase activity and DNA-dependent ATPase activity, with 61.5% (8/13) of genes up-regulated compared to the negative control, vaccinated-and-challenged and vaccinated-only groups. The biological process with the greatest proportion of up-regulated genes per GO term in the challenged-only group was DNA strand elongation, which is involved in DNA replication, with 72.7% (8/11) of genes up-regulated compared to the negative control and vaccinated-only groups and 63.6% (7/11) of genes up-regulated compared to the vaccinated-and-challenged group.

### Gene Ontologies Enriched with Down-Regulated Genes Are Mainly Associated With Impaired Formation and Movement of Cilia

Gene ontology enrichment analysis of down-regulated genes in the challenged-only group identified 33 cellular components, 5 molecular functions, and 42 biological processes enriched with down-regulated genes compared to the negative control group, 35 cellular components, 11 molecular functions, and 46 biological processes enriched with down-regulated genes compared to the vaccinated-and-challenged group, and 40 cellular components, 10 molecular functions, and 52 biological processes enriched with down-regulated genes compared to the vaccinated-only group. The REVIGO non-redundant functional GO summaries of the challenged-only group compared to the negative control, vaccinated-and-challenged, and vaccinated-only groups had similar profiles in the Cellular Component, Molecular Function, and Biological Process GO categories. Briefly, cellular components enriched with down-regulated genes included those involved in the microtubule cytoskeleton, the location of cilia, such as the apical region of the cell, cell projections and the ciliary part, and intraciliary transport particle B, which transports proteins synthesized in the cytoplasm to the axoneme of the cilia (**Figure S4**). The molecular functions enriched with down-regulated genes were those for microtubule motor activity and dynein heavy chain binding (**Figure S5**). The biological processes enriched with down-regulated genes included cell projection assembly and microtubule-based movement, suggesting down-regulation of formation and motor movement of the cilia, and left/right pattern formation and protein complex localization, probably suggesting impaired cell signaling due to disintegration of the epithelium (**Figure S6**). The cellular component with the greatest proportion of down-regulated genes per GO term in the challenged-only group was the axonemal dynein complex, with 78.6% (11/14) of genes down-regulated compared to both the negative control and the vaccinated-and-challenged groups and 85.7% (12/14) of genes down-regulated compared to the vaccinated-only group. Dynein heavy chain binding was the molecular function with the greatest proportion of down-regulated genes in the challenged-only group, with 53.85% (7/13) of genes down-regulated compared to both the negative-control and vaccinated-and-challenged groups and 61.5% (8/13) of genes down-regulated compared to the vaccinated-only group. The biological process with the greatest proportion of down-regulated genes in the challenged-only group was epithelial ciliary movement, with 80% (8/10) of genes down-regulated compared to both the negative control and vaccinated-and-challenged groups and 90% (9/10) of genes down-regulated compared to the vaccinated-only group.

### Pathways Involved in Immune Responses, Cell Proliferation and Signal Transduction Were Activated in the Challenged-Only Group

**Figure 3:**
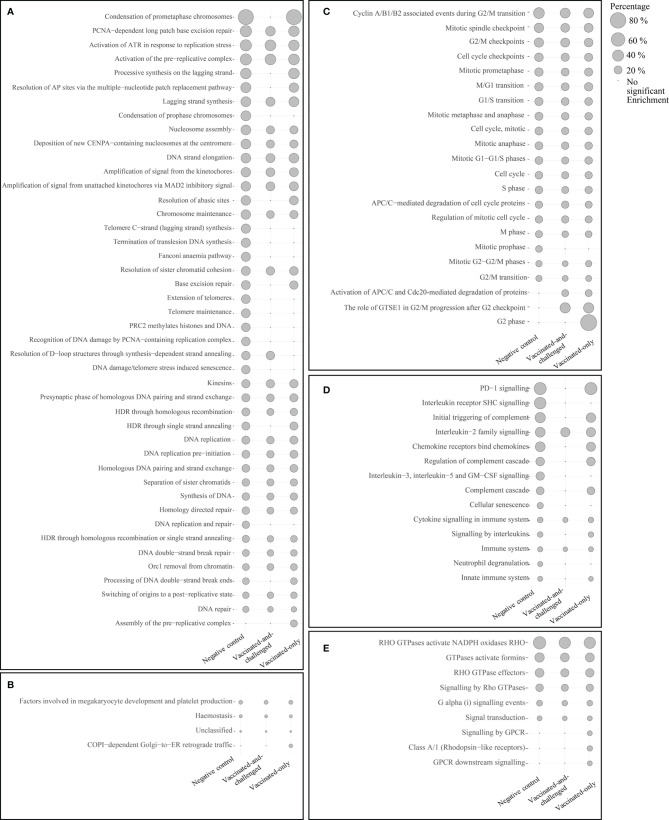
Reactome pathways enriched with up-regulated genes in the challenged-only group compared to the negative control, vaccinated-and-challenged or vaccinated-only group. **(A)** Pathways involved in DNA replication, **(B)** other pathways, **(C)** pathways involved in the cell cycle (mitosis), **(D)** pathways involved in immune responses, **(E)** pathways involved in signal transduction. Grey solid circles indicate the proportion of up-regulated genes out of the total number of genes in each pathway. The pathways are listed in order of decreasing proportions in the negative control group. A false discovery rate (FDR) of < 0.01 was considered significant.

The number of Reactome pathways enriched with up-regulated genes in the challenged-only group compared to the negative control group was 86 (**Figure 3**), with a log_2_ fold enrichment of ≥ 1.82. There were 58 Reactome pathways (**Figure 3**) enriched with up-regulated genes compared to the vaccinated-and-challenged group, with a log_2_ fold enrichment of ≥ 1.96, while there were 77 Reactome pathways (**Figure 3**) enriched with up-regulated genes compared to the vaccinated-only group, with a log_2_ fold enrichment of ≥ 2.00. There were 44 pathways involved in DNA replication (**Figure 3A**), 22 pathways involved in the mitotic cell cycle (**Figure 3C**), 14 pathways involved in immune responses (**Figure 3D**), nine pathways involved in signal transduction (**Figure 3E**), and four other pathways (**Figure 3B**) enriched with up-regulated genes in the challenged-only group compared to one or more other groups.

### Pathways Involved in Formation of Cilia and Transcription Were Down-Regulated in the Challenged-Only Group

**Figure 4:**
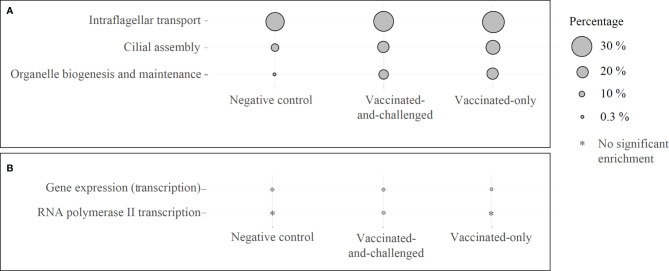
Reactome pathways enriched with down-regulated genes in the challenged-only group compared to the negative control, vaccinated-and-challenged or vaccinated-only group. **(A)** Pathways involved in cilial formation, **(B)** pathways involved in transcription. Gray solid circles indicate the proportion of down-regulated genes out of the total number of genes in each pathway. A false discovery rate (FDR) of < 0.01 was considered significant.

Four reactome pathways were down-regulated in the challenged-only group compared to the negative control group (**Figure 4**), with a log_2_ fold enrichment of ≥ 0.11, while 5 were down-regulated compared to the vaccinated-and-challenged group (**Figure 4**), with a log_2_ fold enrichment of ≥ 0.06. Four pathways were enriched with down-regulated genes compared to the vaccinated-only group, with a log_2_ fold enrichment of ≥ 0.11. Three pathways involved in formation of cilia (**Figure 4A**), and two pathways involved in transcription were down-regulated in the challenged-only group compared to one or more other groups (**Figure 4B**).

### Genes Encoding Protein Classes Involved in Immune Responses and Cell Proliferation Were Up-Regulated While Those for Nucleic Acid Binding Proteins and Cytoskeletal Proteins Were Down-Regulated

Panther protein class analysis of up- and down-regulated genes identified the protein classes enriched at a FDR of < 0.01 (**Table 5**). Six protein classes were enriched with up-regulated genes in the challenged-only group compared to the negative control group, with a log_2_ fold enrichment of ≥ 1.99, six protein classes were enriched with up-regulated genes compared to the vaccinated-and-challenged group, with a log_2_ fold enrichment of ≥ 2.03, and eight classes were enriched with up-regulated genes compared to the vaccinated-only group, with a log_2_ fold enrichment of ≥ 2.07. Protein classes that play a role in immune responses were more highly transcribed in the challenged-only group, mainly cytokines, chemokines, cytokine receptors, Ig receptors and MHC antigens and signaling molecules, as well as replication origin-binding proteins. Three protein classes were enriched with down-regulated genes in the challenged-only group compared to the negative control group, with a log_2_ fold enrichment of ≥ 0.25, five classes were enriched with down-regulated genes compared to the vaccinated-and-challenged group, with a log_2_ fold enrichment of ≥ 0.08, and four classes were enriched with down-regulated genes compared to the vaccinated-only group, with a log_2_ fold enrichment of ≥ 0.06. Those down-regulated protein classes included mainly cytoskeletal proteins and RNA and DNA binding proteins.

**Table 5:** False discovery rates (FDR) of protein classes enriched with up- and down-regulated genes in the challenged-only group compared to the other groups.

	Challenged-only group compared to:		
	Negative control	Vaccinated-and-challenged	Vaccinated-only
**Up-regulated genes**			
Defence/immunity protein	1.08E-07	1.41E-07	1.86E-07
Cytokine	3.26E-05	1.13E-03	3.70E-05
Immunoglobulin receptor superfamily	1.61E-03	7.31E-04	3.40E-03
Signaling molecule	3.22E-03	9.17E-03	2.52E-03
Chemokine	1.13E-03		2.88E-03
Major histocompatibility complex antigen	3.63E-03		3.19E-03
Microtubule binding motor protein		4.32E-03	4.89E-03
Replication origin binding protein		8.09E-03	
Cytokine receptor			7.25E-03
			
**Down-regulated genes**			
Nucleic acid binding	5.21E-07	8.21E-09	7.46E-08
Microtubule family cytoskeletal protein	7.45E-07	3.07E-10	1.02E-10
Cytoskeletal protein	2.57E-06	4.53E-09	5.45E-08
RNA binding protein		1.19E-03	3.75E-04
DNA binding protein		4.06E-03	

A false discovery rate (FDR) of < 0.01 was considered significant.

## Discussion

This study explored the transcriptional profile of the tracheal mucosa of chickens after vaccination with the ts-304 live attenuated vaccine against *M. gallisepticum* and/or infection with virulent *M. gallisepticum*. Comparisons of host gene transcription in the tracheal mucosa of the vaccinated-only, vaccinated-and-challenged, challenged-only and negative control groups at 57 weeks after vaccination identified no differences in gene transcription between the vaccinated-only, vaccinated-and-challenged and negative control groups, but did identify differences in gene transcription between the challenged-only group and every other group, indicating that the vaccine offered a solid protection against gene transcriptional changes in the tracheal mucosa after infection. The different enrichment analyses also suggested that the pathological changes in unvaccinated older birds were similar to, but less severe than, those seen in unvaccinated younger birds after infection in our previous study (28).

Live attenuated vaccines are used to control infection with *M. gallisepticum* and reduce production losses associated with this pathogen in poultry. An ideal live attenuated vaccine is stable, avirulent, affordable, not transmissible, easy to administer and capable of inducing life-long protection (12). Although each of the currently available live attenuated vaccines against *M. gallisepticum*, F strain, 6/85 and ts-11, has its own advantages, none of them is ideal in every respect (12). Therefore, development of vaccines with improved characteristics is necessary to further reduce the impact of losses associated with *M. gallisepticum* in poultry. The findings in this study and our recently reported duration of immunity study (29) have shown that the pathological changes in the tracheal mucosa caused by infection with virulent *M. gallisepticum* are not detectable in birds challenged 57 weeks after vaccination with strain ts-304, demonstrating that the protection induced by this new vaccine maintains the structural and functional integrity of the tracheal mucosa in the face of challenge with an aerosol of virulent *M. gallisepticum*. Assessment of the global transcriptional profiles of the tracheal mucosae of vaccinated-and-challenged birds and comparisons with uninfected birds demonstrated that they had completely recovered from any effects of infection on gene transcription within 2 weeks. This is in agreement with morphometric assessments of the tracheal mucosa and examination of the air sacs for lesions (29). Thus, these studies have shown that the ts-304 vaccine either induces a long-lived immune response that prevents infection or primes the respiratory tract to initiate a rapid anamnestic response against *M. gallisepticum* infection, resulting in protection that extends to the end of the bird’s life in most production systems.

Previous studies examining the efficacy and safety of mycoplasma vaccines have focused on assessment of gross and histopathological lesions and evaluation of the adaptive immune response by measuring the antibody response in vaccinated birds after challenge (11, 16, 22–24, 44, 45). Only a few, recent studies have investigated the genes and/or pathways underlying protection against disease by examining the host responses to vaccination and/or infection with *M. gallisepticum* (26–28) in order to further understand the pathogenetic processes underlying the pathology and the immune responses to infection with the aim of improving the vaccine development process. While the study reported here demonstrated that chickens vaccinated with ts-304 were protected against the adverse effects on the tracheal mucosa after infection with virulent *M. gallisepticum*, we were unable to detect genes or pathways that were involved in this protection, as there were no detectable differences in gene transcription between the two vaccinated groups (vaccinated-and-challenged and vaccinated-only) and the uninfected, negative control group, suggesting that within 2 weeks of infection the immunity provided by the vaccine facilitated the resumption of the normal structural and functional capacity of the tracheal mucosa. Studies investigating the genes involved in the tracheal response shortly after inoculation with attenuated strains of *M. gallisepticum* (26) have shown a reduced intensity and slowed progression in vaccinated birds of the maladaptive responses associated with infection. Chickens vaccinated with strain ts-304 have been found to have significantly higher numbers of B cell clusters and a significantly greater antibody response than birds vaccinated with strain ts-11 at 5 weeks after vaccination (21). In our recent study we detected high concentrations of serum IgG against *M. gallisepticum* at 40, 48, and 57 weeks after vaccination with strain ts-304. These two findings suggest that the strong antibody response mounted by chickens vaccinated with ts-304 may play a role in its improved efficacy. The very low rate of re-isolation of virulent *M. gallisepticum* from the palatine cleft of vaccinated-and-challenged birds suggests that mucosal antibodies may be blocking attachment of *M. gallisepticum* to the respiratory epithelium (29). Further investigation of host responses at an early stage after infection may further enhance our understanding of the mechanisms underlying the protection afforded by vaccination with strain ts-304.

This study also identified differences and similarities in the immunopathological changes seen in this study in the tracheal mucosa of mature birds after challenge with *M. gallisepticum* and those seen in young birds after challenge in a previous study (28). These similarities and differences are outlined in **Table 6**. Notably, the inflammatory response seen in the tracheal mucosa was less severe in birds infected with *M. gallisepticum* at 60 weeks of age than that seen after infection at 7 weeks of age (28) (**Table 6**). An earlier study that investigated the age-related differences in the immune response to infection with *M. gallisepticum* in chickens between 1 and 6 weeks of age found that the lesions were most severe in the younger birds (59), suggesting that older birds are less susceptible to disease caused by infection with *M. gallisepticum*. The inflammatory response in the trachea has more recently been shown to result from the immune dysregulation caused by *M. gallisepticum* (26, 27, 46, 47, 60) and similar transcriptional changes in genes involved in a robust inflammatory response have been detected after infection with *M. ovipneumoniae* in sheep (61, 62) and *M. hyopneumoniae* in pigs (63, 64). The coincidental development of enhanced, macrophage-driven phagocytosis and of the humoral response (**Table 6**) towards the end of the second week after infection suggests the establishment of a protective immune response from this time point on in unvaccinated birds, after an acute phase of immune dysregulation. There were no indications in this study of damage to the apical intercellular junctional complexes in the mature birds, a feature of the changes seen in young birds (28), but the effect of infection with *M. gallisepticum* on the formation and motor movement of cilia and on the formation of the cellular cytoskeleton was similar in both young and mature birds (28) (**Table 6**), suggesting that the effect of infection on the cilia and cytoskeleton of tracheal epithelial cells is independent of the age of the birds, but that the effects on intercellular junctional complexes may be age dependent. The significant downregulation of genes involved in the formation of cilia and the cytoskeleton and in ciliary beating indicates that the loss of cilia and ciliostasis after infection with *M. gallisepticum* is not just an effect of the loss of intercellular adhesion, but rather is modulated at the transcriptional level, as similar transcriptional changes have been seen in the respiratory tracts of sheep and pigs after infection with mycoplasmas (62–64). Subsequent impaired functioning of the mucociliary apparatus (1), which is a primary mode of defense against respiratory pathogens, may enable these pathogens to withstand mucociliary clearance. The transcriptional changes that we detected in this study concorded with histopathological changes, including inflammatory cell infiltration and loss of cilia, that were observed in the tracheal mucosae of the challenged-only birds (unpublished data).

**Table 6:** Similarities and differences between the immunopathological changes in the tracheal mucosa induced by *M. gallisepticum* in mature birds and young birds (28).

Feature		References
**Similarities**	
**(1) Robust inflammatory response mediated by cytokines, chemokines and TLRs**.		
	Up-regulation of genes for IL-22, CXCL13, CXCL13-like 2, lymphotactin, CCL-19, CCL-26, IFN-γ, chemokine ah221, MIP-1β, IL-16, CXCL13-like 3, CXCL12 (**Table 3**).	(46–47, 48)
	Up-regulation of genes for TLR7, TLR1 family member A and TLR2 family member B (**Table 3**).	(26, 27, 47)
	Enrichment of GOs (**Figures S2-3**), pathways (**Figure 3**) and protein classes (**Table 4**) involved in cytokine/chemokine production, signaling and receptor binding.	
**(2) Ability of *M. gallisepticum* to induce local activation and proliferation of inflammatory cells increasing the mucosal thickness**.		
	Up-regulation of genes and enrichment of GOs (**Figures S1-3**), pathways (**Figure 3**) and protein classes (**Table 4**) involved in DNA replication and cell cycle.	
**(3) Ability of *M. gallisepticum* to suppress TLR5-mediated responses against flagellated bacteria**.		
	Down-regulation of TLR5 (**Table 3**)	(49)
**(4) B cell migration and onset of adaptive immune response 2 weeks after infection following a phase of immune dysregulation**.		
	Up-regulation of genes for B cell chemoattractants including CXCL13, CXCL13-like 2, CXCL13-like 3, lymphotactin and IL-16 (**Table 3**).	(50)
	Increased CXCL12-CXCR4 interaction (**Table 3**).	(51)
	Enrichment of GOs involved in B cell recruitment (**Figure S3**).	
**(5) Macrophage driven phagocytosis**		
	Up-regulation of genes for IFN-γ, MIP-1β, and chemokine ah221 (**Table 3**)	
	Enrichment of GOs (**Figure S3**) involved in phagocytosis	
	Enrichment of pathways involved in phagocytosis including Activation of formins and NADP oxidase by Rho GTPases (**Figure 3**)	
**(6) Impaired formation and motor movement of cilia**		
	Down-regulation of genes (**Table 2**) and enrichment of GOs (**Figures S4-6**), pathways (**Figure 4**) and protein classes (**Table 4**) with down-regulated genes involved in formation and motor movement of cilia, with more effect on anterograde intraflagellar transport mediated by dynein arm	(25, 52–54)
**(7) Impaired formation of cellular cytoskeleton**		
	Down-regulation of genes (**Table 2**) and enrichment of GOs (**Figures S4-6**), pathways (**Figure 4**) and protein classes (**Table 4**) with down-regulated genes involved in formation of cytoskeleton	
**Differences**		
**(1) Decreased severity of inflammation**		
	No significant difference in transcription of genes for IL-17A, IL-1β, IL-6, and IL-8, which are known proinflammatory cytokines	(55, 56)
	No significant difference in the transcription of IL-1 receptor 2, which acts as a “decoy receptor” for IL-1β, decreasing the effective level of IL-1β	(57)
	Enrichment of PD-1 signaling pathway (**Figure 2**) with up-regulated genes. PD-1 signaling pathway reduces inflammation by immunosuppression through inhibition of T cell proliferation, cytokine production and cytolytic function	(58)
		
**(2) No damage to formation of apical intercellular junctional complexes**		

Although the two unvaccinated control (challenged-only and negative control) groups were reared in a different environment and were slightly older than the two vaccinated (vaccinated-only and vaccinated-and-challenged) groups, there were no differences in the global gene transcriptional profiles of the negative control group and the two vaccinated groups, indicating that the transcriptional changes seen in the challenged-only group were specific for infection with virulent *M. gallisepticum*.

In summary, this study has demonstrated that vaccination with the attenuated *M. gallisepticum* strain ts-304 protects against the adverse immune dysregulation in the tracheal mucosa caused by infection with *M. gallisepticum* in chickens for at least 57 weeks after vaccination.

## Data Availability Statement

The data sets presented in this study can be found in online repositories. The names of the repository/repositories and accession number(s) can be found below: https://www.ncbi.nlm.nih.gov/, PRJNA605218.

## Ethics Statement

The animal study was reviewed and approved by the University of Melbourne Animal Ethics Committee under approval number 1513791.3.

## Author Contributions

GB and NW designed the study and prepared the study protocol. AKC, SKA, NW, OO, and AN performed the challenge studies and necropsies. SKA carried out the RNA extractions and cDNA library preparation. SKA and NY performed data curation and analysis. SKA wrote the original draft and all other authors contributed to preparation of the manuscript. All authors contributed to the article and approved the submitted version.

## Funding

This study was supported by the Australian Research Council through Linkage Project grant LP160101105 in partnership with Bioproperties Pty. Ltd.

## Conflict of Interest

The University of Melbourne licenses the *M. gallisepticum* ts-304 vaccine to Bioproperties Pty. Ltd. and, as employees of the university involved in the creation of ts-304, AKC and GB are entitled to a share of any royalties generated from this license.

The authors declare that this study received funding from Bioproperties Pty. Ltd. The funder had involvement in the design of the experimental infection component of the study.

The remaining authors declare that the research was conducted in the absence of any commercial or financial relationships that could be construed as a potential conflict of interest.
