# Supplementary material for: Transcriptomic Analysis of Long-Term Protective Immunity Induced by Vaccination With Mycoplasma gallisepticum Strain ts-304
Source: Front Immunol. 2021 Feb 2;11:628804. doi: 10.3389/fimmu.2020.628804 (PMC7885271; doi:10.3389/fimmu.2020.628804)
Supplement: Supplementary file 1 [file DataSheet_1.docx]

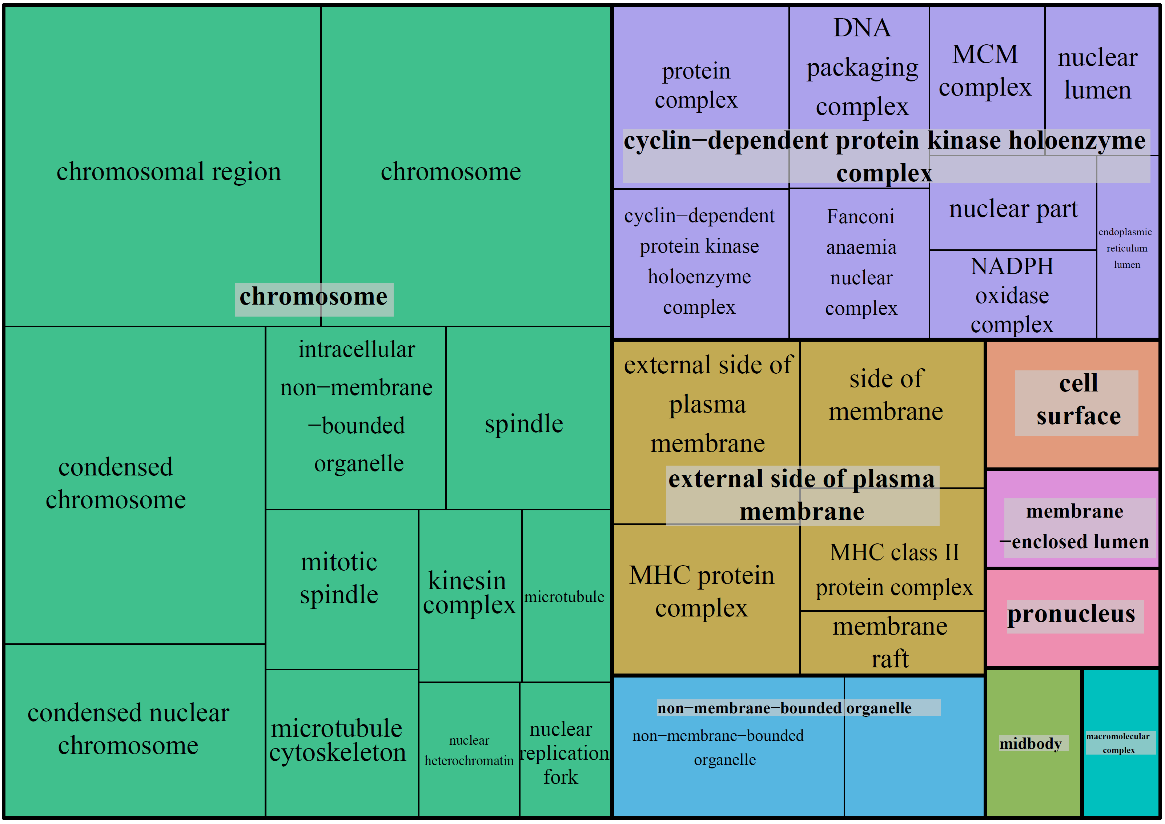


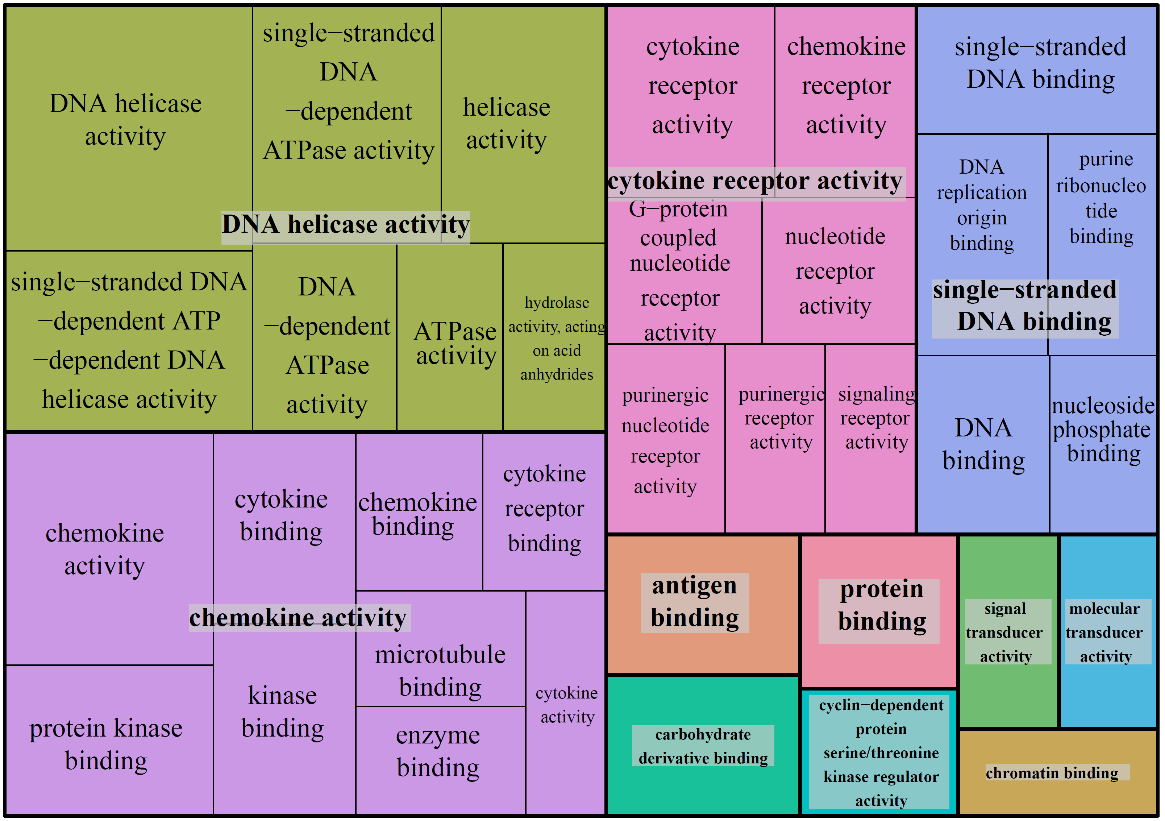
**Fig S1.** Groups (differentiated by color) of upregulated cellular components in the challenged-only group compared to the negative control group based on the summarised GOs. The sizes of the boxes are directly proportional to the significance, with a FDR of < 0.01 considered significant.

**Fig S2.** Groups (differentiated by color) of upregulated molecular functions in the challenged-only group compared to the negative control group based on the summarised GOs. The sizes of the boxes are directly proportional to the significance, with a FDR of < 0.01 considered significant.


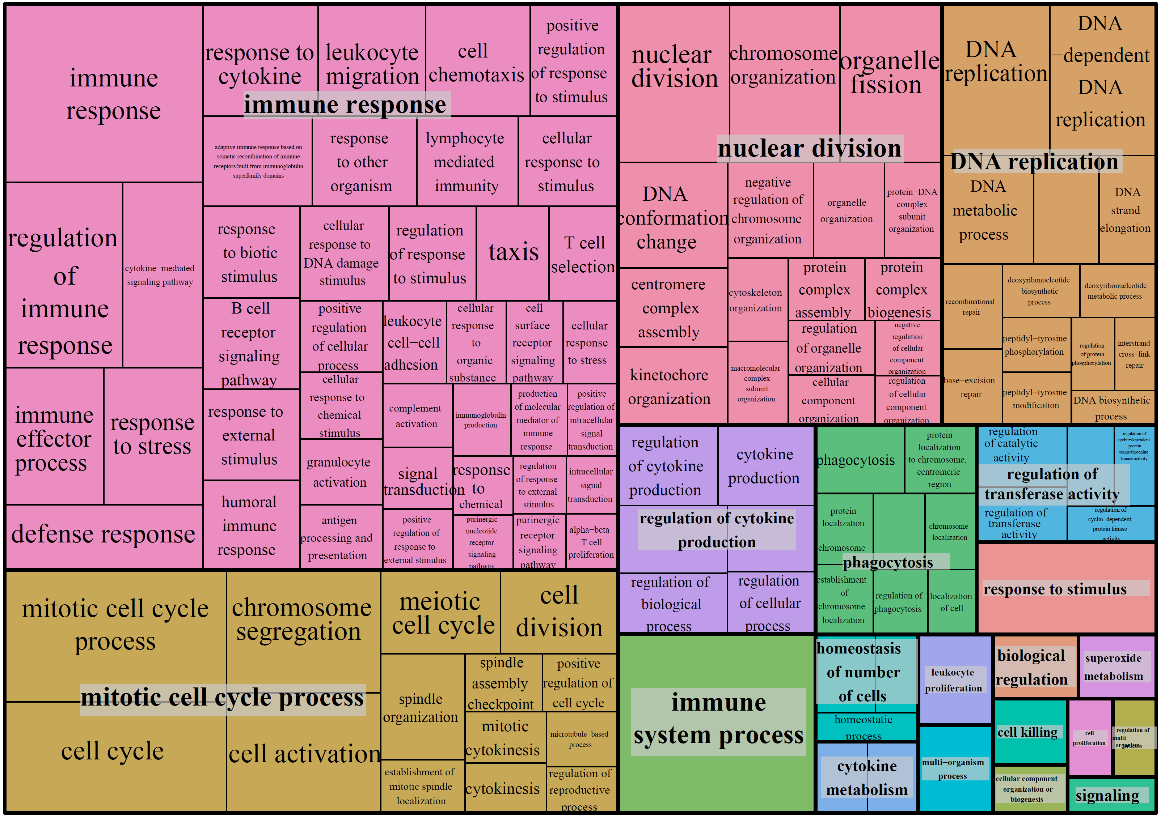


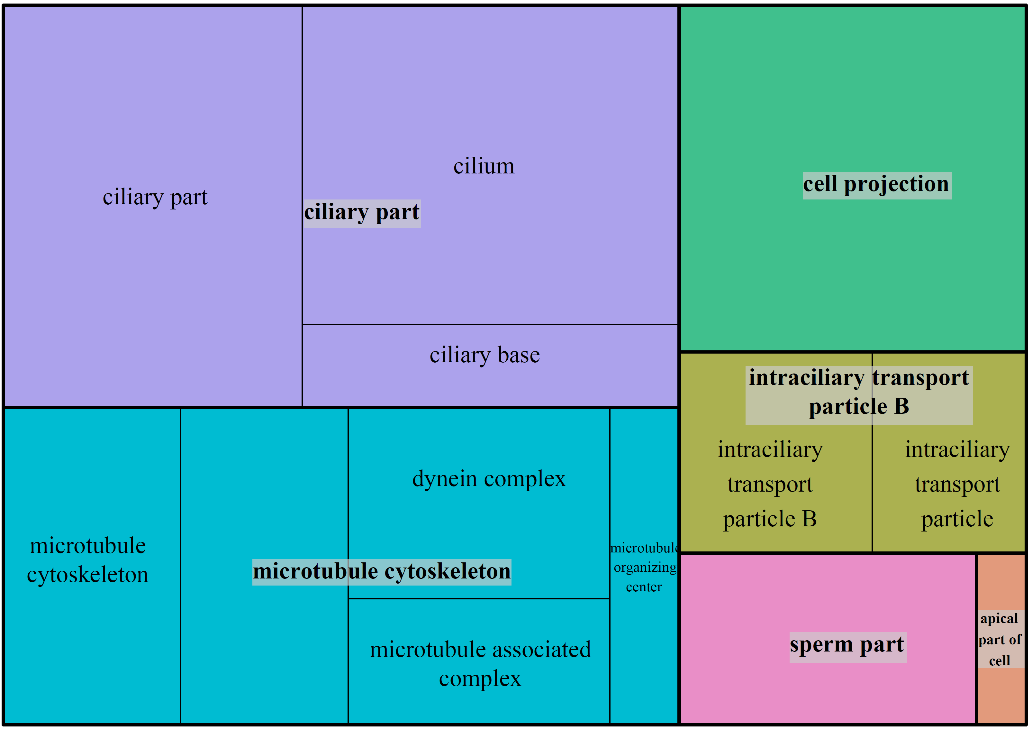
**Fig S3.** Groups (differentiated by color) of upregulated biological processes in the challenged-only group compared to the negative control group based on the summarised GOs. The sizes of the boxes are directly proportional to the significance, with a FDR of < 0.01 considered significant.

**Fig S4.** Groups (differentiated by color) of downregulated cellular components in the challenged-only group compared to the negative control group based on the summarised GOs. The sizes of the boxes are directly proportional to the significance, with a FDR of < 0.01 considered significant.


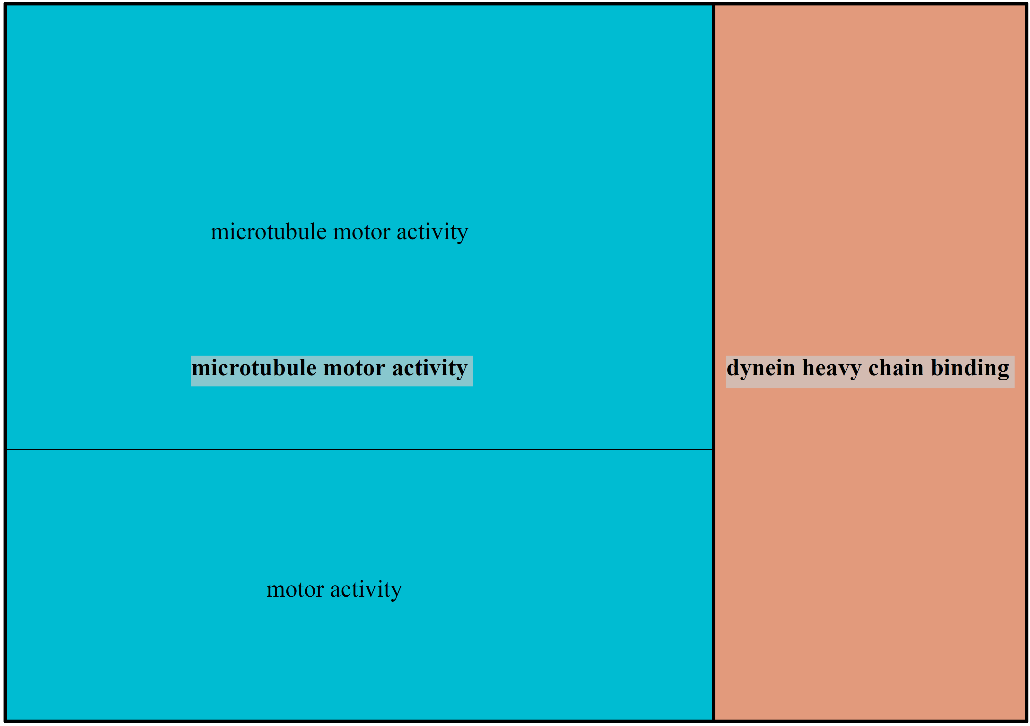


**Fig S5.** Groups (differentiated by color) of downregulated molecular functions in the challenged-only group compared to the negative control group based on the summarised GOs. The sizes of the boxes are directly proportional to the significance, with a FDR of < 0.01 considered significant.


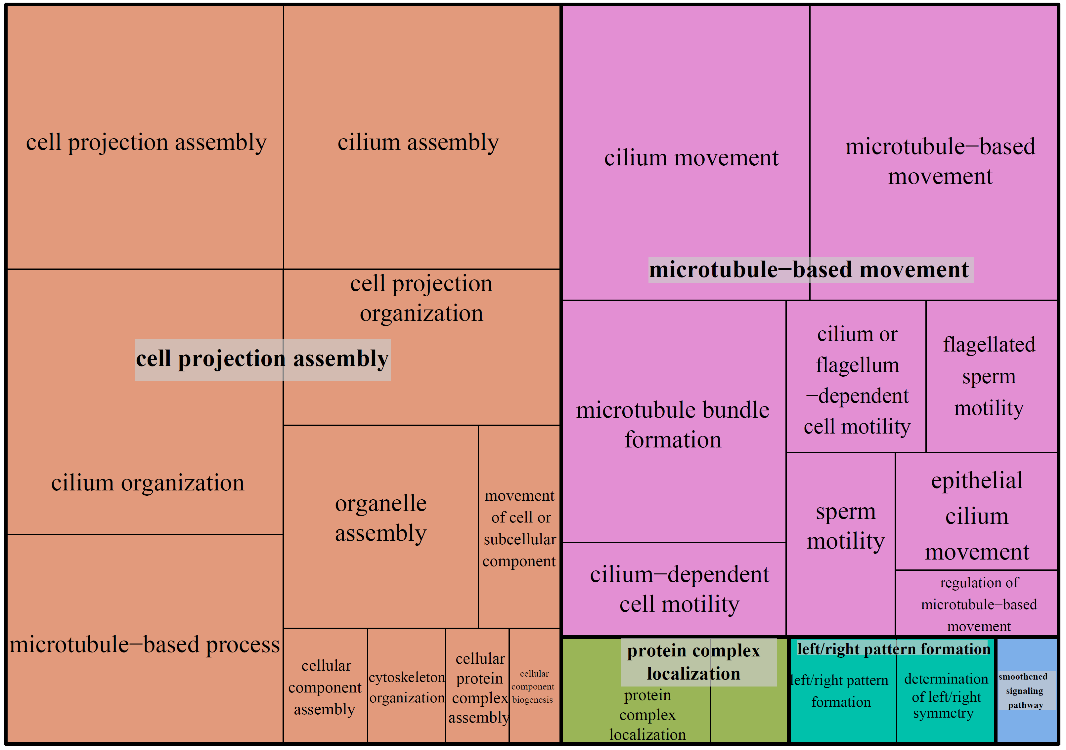


**Fig S6.** Groups (differentiated by color) of downregulated biological processes in the challenged-only group compared to the negative control group based on the summarised GOs. The sizes of the boxes are directly proportional to the significance, with a FDR of < 0.01 considered significant.
